# circKIF4A acts as a prognostic factor and mediator to regulate the progression of triple-negative breast cancer

**DOI:** 10.1186/s12943-019-0946-x

**Published:** 2019-02-11

**Authors:** Hailin Tang, Xiaojia Huang, Jin Wang, Lu Yang, Yanan Kong, Guanfeng Gao, Lijuan Zhang, Zhe-Sheng Chen, Xiaoming Xie

**Affiliations:** 1 Department of Breast Oncology, Sun Yat-sen University Cancer Center, State Key Laboratory of Oncology in South China, Collaborative Innovation Center of Cancer Medicine, 651 East Dongfeng Road, Guangzhou, 510060 China; 2 Department of Pharmaceutical Sciences, College of Pharmacy and Health Sciences, St. John’s University, Queens, New York, 11439 USA

**Keywords:** Circular RNAs, miR-375, KIF4A, Competitive endogenous RNAs, Triple negative breast cancer

## Abstract

**Background:**

Increasing studies has found that circular RNAs (circRNAs) play vital roles in cancer progression. But the expression profile and function of circRNAs in triple-negative breast cancer (TNBC) are unclear.

**Methods:**

We used a circRNA microarray to explore the circRNA expression profile of TNBC. The expression of the top upregulated circRNA, circKIF4A, was confirmed by qRT-PCR in breast cancer cell lines and tissues. Kaplan-Meier survival analysis was conducted to analyze the clinical impact of circKIF4A on TNBC. A series of experiments was performed to explore the functions of circKIF4A in TNBC progression, such as cell proliferation and migration. We investigated the regulatory effect of circKIF4A on miRNA and its target genes to explore the potential regulatory mechanisms of circKIF4A in TNBC.

**Results:**

qRT-PCR analyses verified that circKIF4A was significantly upregulated and positively associated with poorer survival of TNBC. The inhibition of circKIF4A suppressed cell proliferation and migration in TNBC. Luciferase reporter assay and RNA immunoprecipitation assay revealed that circKIF4A and KIF4A could bind to miR-375 and that circKIF4A regulated the expression of KIF4A via sponging miR-375.

**Conclusions:**

The circKIF4A-miR-375-KIF4A axis regulates TNBC progression via the competitive endogenous RNA (ceRNA) mechanism. circKIF4A may therefore serve as a prognostic biomarker and therapeutic target for TNBC.

**Electronic supplementary material:**

The online version of this article (10.1186/s12943-019-0946-x) contains supplementary material, which is available to authorized users.

## Background

Circular RNAs (circRNAs) have multiple functions, including miRNA binding, protein binding, regulation of protein translation [1]. CircRNAs can act as oncogenes or tumor suppressors, therefore, they could be used as biomarkers or therapeutic targets for cancer. But the role of circRNAs in triple-negative breast cancer (TNBC) remains unclear.

RNAs can serve as competitive endogenous RNAs (ceRNAs) and compete for shared microRNAs (miRNAs) [2]. circRNAs have advantages as ceRNAs, as they have no free ends, predominant localization in the cytoplasm and a largely noncoding nature [3]. With miRNA-binding sites, circRNAs could function as miRNA sponges, which would lead to loss of miRNA function and which would be accompanied by increased gene targets. Memczak S et al. found that the circRNA CDR1as was a negative regulator of miR-7 [4]. In cancers, the circRNA CCDC66 has been shown to sponge miRNAs that target oncogenes, which promote cancer growth and metastasis [5]. Moreover, circPVT1 also sponges several tumor suppressor miRNAs including let-7b [6]. These findings have urged us to explore the use of circRNAs as therapeutic targets.

Here, we reanalyzed the circRNA expression microarray that we did in our previous study [7]. The top upregulated circRNA termed circKIF4A was confirmed significantly upregulated and positively associated with a worse outcome in TNBC. A series of in vitro and in vivo experiments was performed to explore the function of circKIF4A in TNBC progression. We found that circKIF4A could regulate TNBC cell proliferation and migration. Then we explored the potential regulatory mechanisms of circKIF4A in TNBC and found that circKIF4A regulated the expression of KIF4A via sponging miR-375 to exert its regulatory functions in TNBC. The circKIF4A-miR-375-KIF4A axis regulates TNBC progression via the ceRNA mechanism. Therefore, circKIF4A may act as a prognostic biomarker and therapeutic target for TNBC.

## Methods

### Cell culture and transfection

All the cell lines, including a human mammary epithelial cell line (MCF10A), NTNBC cell lines (MCF-7, T47D, BT474 and SKBR3) and TNBC cell lines (MDA-MB-453, MDA-MB-468, MDA-MB-231, BT549 and HCC38), were purchased from American Type Culture Collection. All the cell lines were free of mycoplasma infection, and DNA fingerprinting was performed to verify cell authenticity.

Cells were transfected with Lipofectamine 2000 (Invitrogen, USA). siRNAs targeting circKIF4A were synthesized by GenePharma (China), the sequences are presented in Additional file 1: Table S1. miR-375 mimics and inhibitors were purchased from GeneCopoeia (USA).

### Quantitative real-time PCR (qRT-PCR)

Total RNA was isolated by TRIzol (Invitrogen) and the nuclear and cytoplasmic fractions were isolated by NE-PER™ Nuclear and Cytoplasmic Extraction Reagents (Thermo Scientific). qRT-PCR was conducted with SYBR Premix Ex Taq™ (Takara, Japan) and an All-in-One™ miRNA qRT-PCR Detection Kit (GeneCopoeia) using Bio-Rad IQTM5 Multicolor Real-Time PCR Detection System (USA). The primers for qRT-PCR were purchased from Invitrogen (Additional file 1: Table S1).

### Cell counting kit-8 (CCK-8) assay

Cells (1 × 10^3^) were seeded into 96-well plates and CCK-8 solution (Dojindo Laboratories, Japan, 10 μl) was added 48 h after transfection. The absorbance at 450 nM was measured after incubation at 37 °C for 2 h with microtiter plate reader (Bio-Tek EPOCH2, USA).

### Colony formation assay

Cells (1 × 10^3^) were seeded in 6-well plates and incubated at 37 °C for 2 weeks. Colonies were fixed in methanol, stained with 0.1% crystal violet, imaged and counted.

### Transwell assay

Transwell assays were performed with migration chambers (BD Biosciences, USA). Briefly, cells (1 × 10^4^) were seeded and medium with 10% FBS was added to the lower chamber as a chemoattractant. After 24 h, cells were fixed in methanol, stained with 0.1% crystal violet and counted.

### Wound-healing assay

Briefly, cells were seeded, and a linear wound was generated within the confluent monolayers by scraping the cells with sterile 1-mL pipette tips. 24 h later the progression of migration was imaged with an inverted microscope.

### Immunofluorescence staining

Cells were seeded and fixed with 4% paraformaldehyde for 20 min, then permeabilized with 0.5% Triton X-100 for 10 min and blocked with 4% bovine serum albumin for 1 h then incubated with primary antibodies of E-cadherin and vimentin (Cell Signaling Technology, USA, 1:100) at 4 °C overnight. Then incubated with Dylight-conjugated secondary antibody (Abbkine, China; 1:200) for 1 h. Antifade DAPI solution (Sigma, USA) was added, and cells were imaged.

### Mouse xenograft model

Cells (2 × 10^6^) were subcutaneously injected into the dorsal flanks of 4-week-old female BALB/c nude mice (five mice per group). Then the mice were intratumorally injected with 40 μL si-NC or si-circKIF4A every 4 days. Xenografts were excised under anesthesia after 4 weeks, and the tumor weights were measured.

For lung metastasis studies, cells (1 × 10^5^) were injected into the mice tail veins (six mice per group). Lung metastasis was monitored by Xenogen IVIS Spectrum Imaging System (PerkinElmer, USA). After 8 weeks, the lungs were excised under anesthesia, and the numbers of macroscopically visible lung metastatic nodules were counted and validated by assessment of hematoxylin and eosin (HE)-stained sections by microscopy.

### Luciferase reporter assay

The circKIF4A sequences including the miR-375 binding sites (AGCAAGAAAAAUCAAGAACAAAC) was inserted into the pGL3 luciferase vector (Promega, USA) immediately downstream of luciferase. Mutations in the miR-375 seed-region were conducted with Fast Site-Directed Mutagenesis Kit (TIANGEN, China). The KIF4A 3’-UTR including the miR-375 binding sites (UGCUGUUGAAAAAAGGAGCAAAG) was inserted into the pGL3 luciferase vector. Mutations in the miR-375 seed-region served as a mutant control.

Cells (5 × 10^3^) were seeded and cotransfected with corresponding vectors and miR-375 mimics or inhibitors. After 48 h of incubation, luciferase intensity was measured by dual-luciferase reporter assay system (Promega).

### RNA immunoprecipitation (RIP) assay

Cells were cotransfected with MS2bs-circKIF4A, MS2bs-circKIF4Amt or MS2bs-Rluc and MS2bp-GFP. After 48 h, RIP was performed with Magna RIP RNA-Binding Protein Immunoprecipitation Kit (Millipore, USA). The RNA complexes were then purified, and the level of miR-375 was quantified.

For the RIP assay for Ago2, RIP was performed with an anti-Ago2 antibody (Millipore). RNAs were then purified, and the levels of circKIF4A, KIF4A and miR-375 were measured.

### Western blot

Briefly, proteins were extracted, quantified and separated by 10% SDS-PAGE and transferred to PVDF membranes (Millipore). Then incubated with 5% skim milk at room temperature for 1 h and with primary antibody of KIF4A (1:100, Abcam, USA). Then incubated with HRP-labeled secondary antibody (CST) and detected by chemiluminescence. Anti-β-actin antibody (11,000, Affinity, USA) was used as a control.

### Statistical analysis

Statistical analysis was conducted with SPSS 19.0 software. Comparisons between groups were performed using t tests and Pearson χ^2^ tests. Kaplan-Meier plots and log-rank tests were used for the survival analysis. Unless otherwise indicated, data are presented as the mean ± S.D. of three independent experiments. *P* < 0.05 was considered statistically significant.

## Results

### circKIF4A is upregulated and correlated with poor clinical outcomes of TNBC

**Fig. 1:**
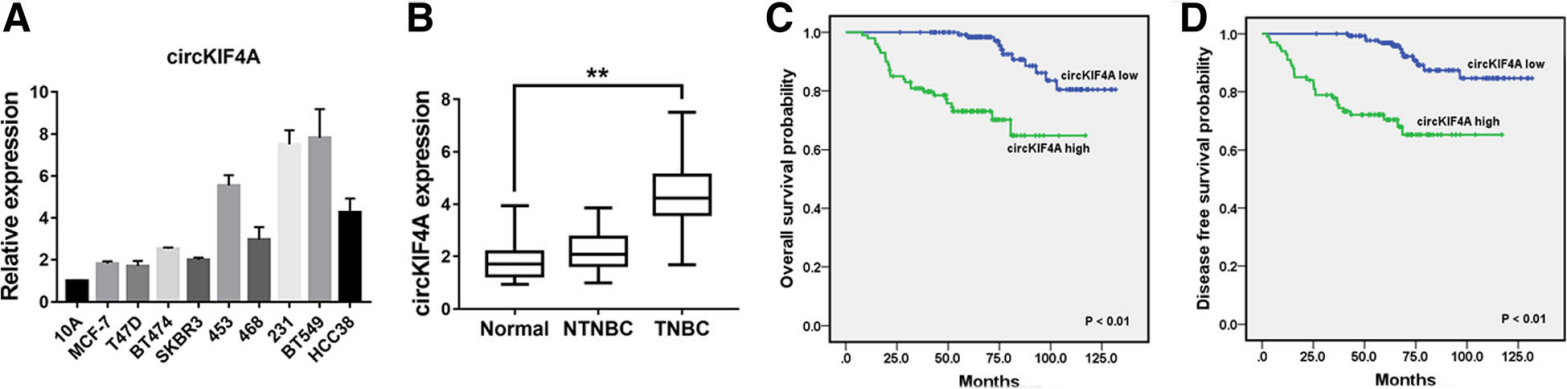
circKIF4A is upregulated and correlated with poor clinical outcomes of TNBC **a** The circKIF4A expression in breast cancer cell lines. **b** The circKIF4A expression in breast cancer tissues and normal adjacent tissues. **c** OS curves for 240 TNBC patients. **d** DFS curves for 240 TNBC patients. **P < 0.01

To explore the circRNA expression profile of TNBC, we reanalyzed the circRNA microarray we did in our previous study [7]. We performed qRT-PCR to verify the expression of the top upregulated circRNA, hsa_circ_0007255, in breast cancer cell lines and tissues. According to the human reference genome (GRCh37/hg19), hsa_circ_0007255 is located at chrX: 69549254–69553539 and is assumed to be derived from the KIF4A (kinesin family member 4A) gene. Therefore, we named hsa_circ_0007255 “circKIF4A”. qRT-PCR showed that circKIF4A was upregulated in TNBC cell lines (Fig. 1a). Next, we detected circKIF4A expression in breast cancer tissues and adjacent normal tissues and found that circKIF4A was significantly upregulated in TNBC tissues (Fig. 1b).

To investigate the clinical significance of circKIF4A in TNBC, a cohort of 240 TNBC patients was recruited. Patients who expressed circKIF4A equal to or greater than the average level were assigned to the “circKIF4A high” group. Our analysis showed that circKIF4A was positively associated with tumor size, lymph node metastasis and TNM stage (Table 1), which indicates that circKIF4A plays a vital role in TNBC progression. Next, we performed Kaplan-Meier survival analysis and found that circKIF4A was positively associated with poorer survival of patients with TNBC (Fig. 1c and d).

**Table 1:** Association between circKIF4A and the clinicopathological characteristics of TNBC

Variables	Cases (*n* = 240)	circKIF4A		*P* value
		Low (*n* = 140)	High (*n* = 100)	
Age (years)				0.078
< 50	136	86 (63.2%)	50 (36.8%)	
≥ 50	104	54 (51.9%)	50 (48.1%)	
Menopause				0.109
No	144	90 (62.5%)	54 (37.5%)	
Yes	96	50 (52.1%)	46 (47.9%)	
Tumor Size				<0.001*
≤ 2.0 cm	66	51 (77.3%)	15 (22.7%)	
> 2.0 cm	174	89 (51.1%)	85 (48.9%)	
Lymph node Metastasis				<0.001*
No	123	86 (69.9%)	37 (30.1%)	
Yes	117	54 (46.2%)	63 (53.8%)	
TNM Stage				0.012*
I-II	187	117 (62.6%)	70 (37.4%)	
III-IV	53	23 (43.4%)	30 (56.6%)	

*P < 0.05, statistically significant

### Knockdown of circKIF4A inhibits proliferation and metastasis of TNBC

**Fig. 2:**
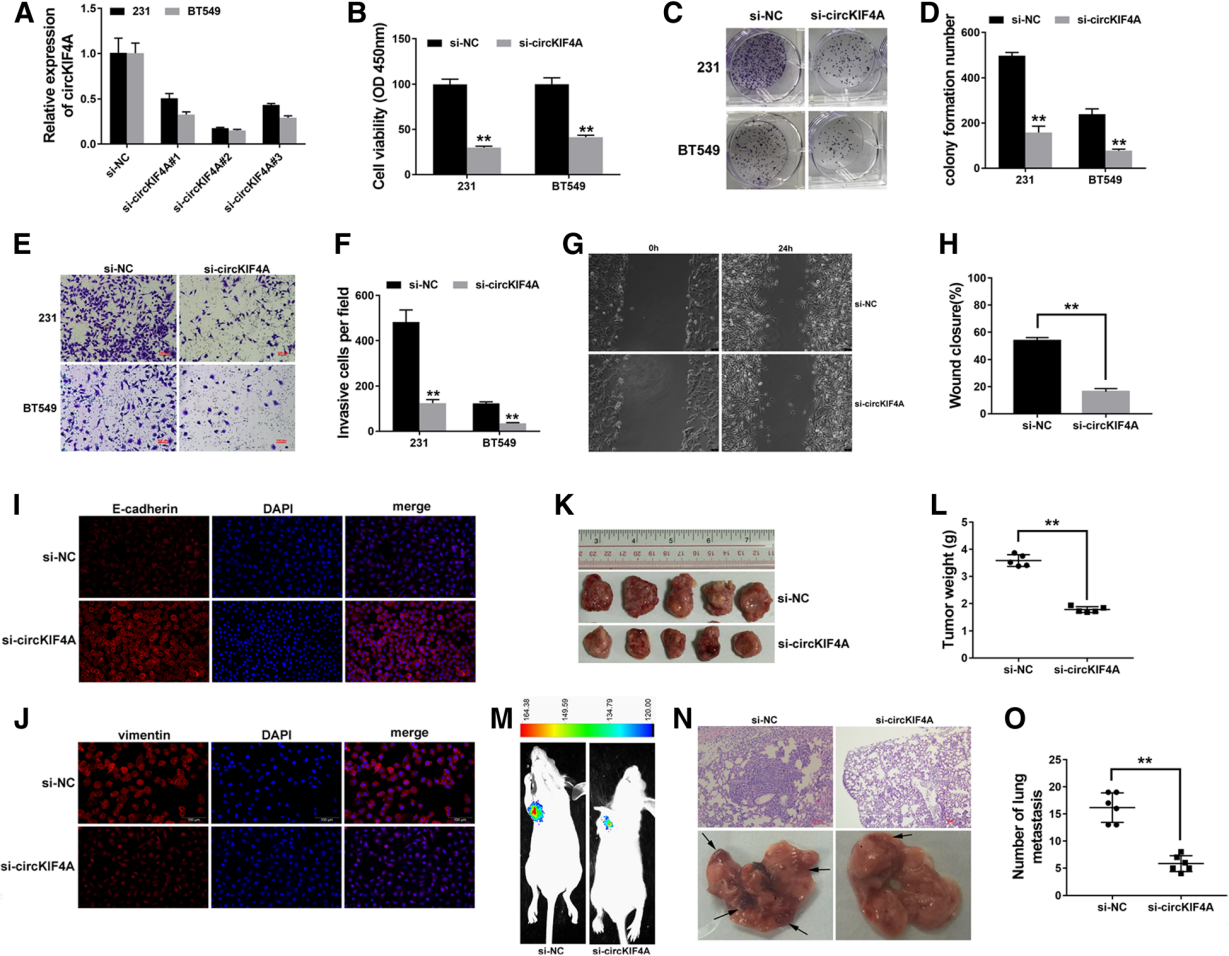
Knockdown of circKIF4A inhibits proliferation and metastasis of TNBC **a** si-circKIF4A #2 successfully knocked down circKIF4A. **b** A CCK-8 assay to detect cell proliferation. **c** A colony formation assay to detect cell colony-forming ability. **d** Colony formation number was quantified by ImageJ. **e** A Transwell assay to assess cell migratory ability. **f** The number of invasive cells was quantified by ImageJ. **g** A wound-healing assay to assess cell migratory capability. **h** Wound closure was quantified by ImageJ. **i-j** Immunofluorescence staining of E-cadherin and vimentin. **k** Xenograft models were established. **l** Summary of tumor weights. **m** Representative image of luciferase signals of lung metastatic nodules. **n** Representative images of lung metastatic nodules and HE-stained sections. **o** The number of metastatic nodules was quantified. **P < 0.01

To explore the function of circKIF4A in TNBC, we knocked down circKIF4A expression; the inhibition was successful with si-circKIF4A#2, which was used in the following experiments (Fig. 2a). A CCK-8 assay showed that circKIF4A knockdown significantly inhibited cell proliferation (Fig. 2b). circKIF4A knockdown also reduced the colony formation ability of the cells (Fig. 2c and d). Transwell assay revealed that cell metastasis was significantly reduced after downregulation of circKIF4A (Fig. 2e and f). Wound-healing assay also revealed that knockdown of circKIF4A significantly suppressed cell migration capability (Fig. 2g and h). Immunofluorescence staining revealed that knockdown of circKIF4A increased the expression of the epithelial marker E-cadherin, while it decreased the expression of the mesenchymal marker vimentin (Fig. 2i and j). To further explore the function of circKIF4A in vivo, mouse xenograft models were established. circKIF4A inhibition significantly decreased tumor growth (Fig. 2k and l) and lung metastasis (Fig. 2m-o), which indicates that the knockdown of circKIF4A inhibits cell proliferation and metastasis in TNBC.

### circKIF4A acts as a sponge for miR-375

**Fig. 3:**
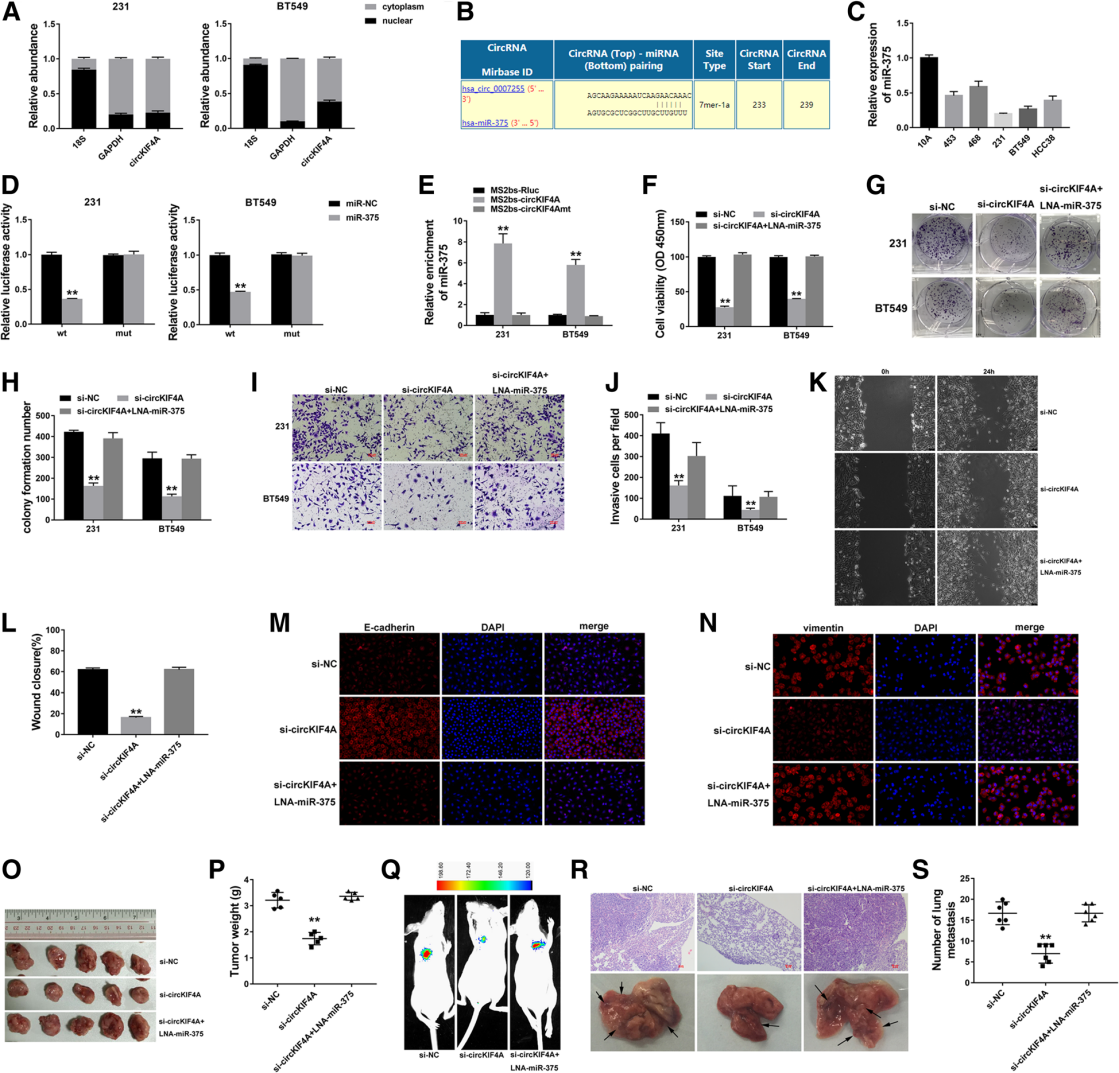
circKIF4A acts as a sponge for miR-375 **a** The expression levels of nuclear control (18S), cytoplasmic control (GAPDH) and circKIF4A were detected. **b** The predicted binding sites of miR-375 within the circKIF4A sequence. **c** The miR-375 expression in TNBC cell lines. **d** Luciferase assay of cells cotransfected with miR-375 mimics and wild type or mutant luciferase reporter. **e** MS2-based RIP assay transfected with MS2bs-circKIF4A, MS2bs-circKIF4Amt or control. **f** A CCK-8 assay to detect cell proliferation. **g** A colony formation assay to detect cell colony-forming ability. **h** The colony formation number was quantified by ImageJ. **i** A Transwell assay to assess cell migration ability. **j** The number of invasive was quantified by ImageJ. **k** A wound-healing assay to detect cell migration ability. **l** Wound closure was quantified by ImageJ. **m-n** Immunofluorescence staining for E-cadherin and vimentin. **o** Xenograft models were established. **p** Summary of the tumor weights. **q** Representative image of luciferase signals of lung metastatic nodules. **r** Representative images of lung metastatic nodules and HE-stained sections. **s** The number of metastatic nodules was quantified. **P < 0.01

We detected the intracellular location of circKIF4A and found that this circRNA was predominantly localized in the cytoplasm (Fig. 3a), which indicates that it might function as a miRNA sponge. Therefore, we used Circular RNA Interactome (https://circinteractome.nia.nih.gov/index.html) to predict the potential circRNA/miRNA interaction. In addition, binding sites for miR-375 were found within the circKIF4A sequence (Fig. 3b). We detected the expression of miR-375 in TNBC cell lines and miR-375 was downregulated (Fig. 3c). A subsequent luciferase reporter assay revealed that the luciferase intensity was reduced after the cotransfection of the wild type luciferase reporter and miR-375 mimics, while the mutated luciferase reporter exerted no such effect (Fig. 3d). To confirm the direct binding of circKIF4A and miR-375, a RIP assay was performed. The result revealed that miR-375 was predominantly enriched in MS2bs-circKIF4A group (Fig. 3e), which indicates that circKIF4A directly interacts with miR-375 and could act as a sponge for miR-375.

To validate that circKIF4A functions through sponging miR-375, a series of rescue experiments was performed. CCK-8 and colony formation assays revealed that cell proliferation suppression induced by circKIF4A downregulation was reversed by treatment with a miR-375 inhibitor (Fig. 3f-h). Transwell and wound-healing assays revealed that reduced cell migration ability caused by circKIF4A downregulation was reversed by treatment with a miR-375 inhibitor (Fig. 3i - l). Immunofluorescence staining demonstrated that the expression changes in E-cadherin and vimentin caused by circKIF4A knockdown were reversed by treatment with a miR-375 inhibitor (Fig. 3m and n). Experiments using the xenograft mouse model showed that decreased tumor growth and lung metastasis after inhibition of circKIF4A were reversed by treatment with a miR-375 inhibitor (Fig. 3o - s). All these findings suggest that circKIF4A functions through sponging miR-375 in TNBC.

### circKIF4A acts as a ceRNA to regulate KIF4A

**Fig. 4:**
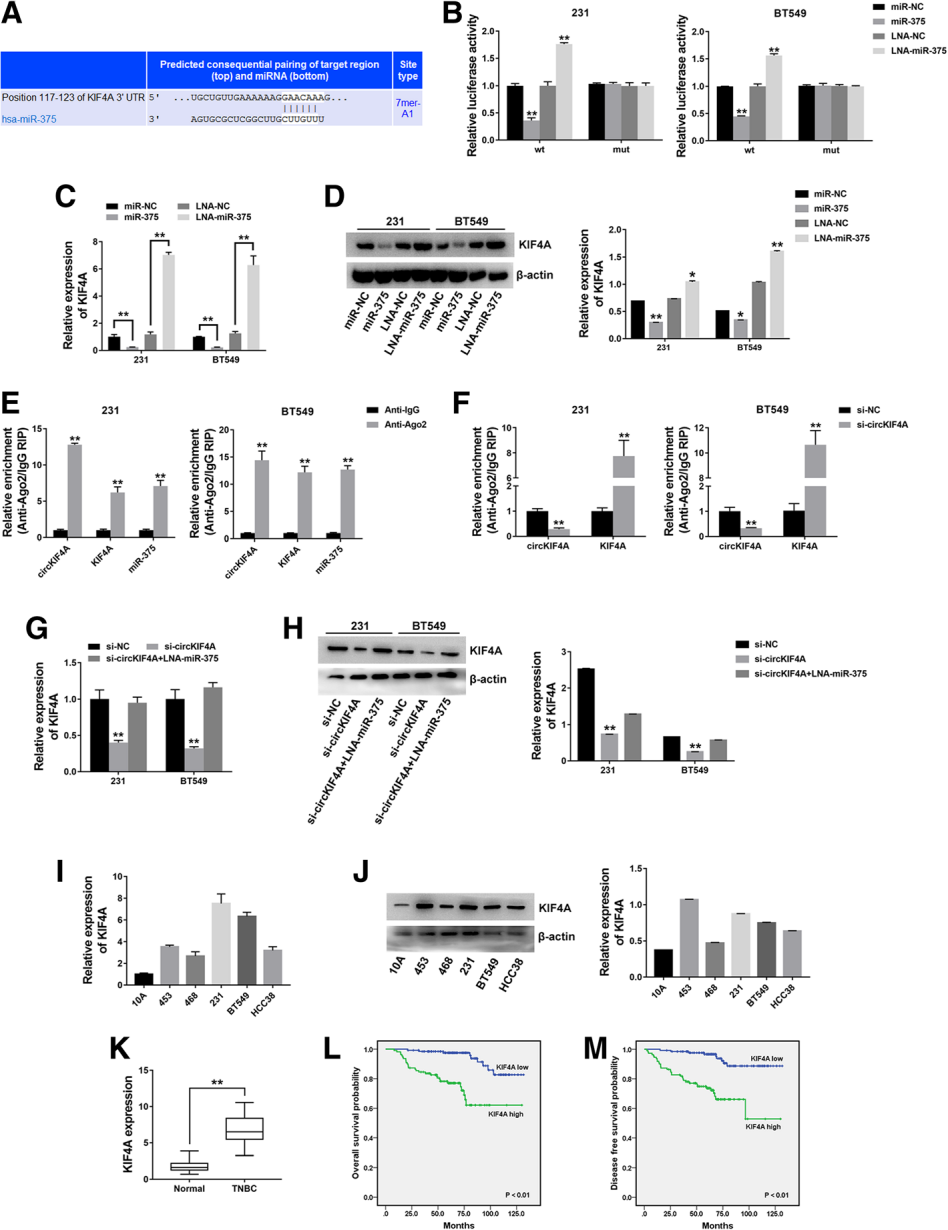
circKIF4A acts as a ceRNA to regulate KIF4A **a** The predicted binding sites of miR-375 within the KIF4A 3’UTR. **b** Cells were transfected and luciferase assay was performed. **c** Cells were transfected and KIF4A expression was detected by qRT-PCR. **d** The KIF4A expression was detected by western blot (left) and quantified (right). **e** RIP assay showing the enrichment of circKIF4A, KIF4A and miR-375 on Ago2. **f** Cells were transfected and a RIP assay on Ago2 was performed. **g** Cells were transfected and KIF4A expression was detected by qRT-PCR. **h** KIF4A expression was detected by western blot (left) and quantified (right). **i** The KIF4A expression in TNBC cell lines was detected by qRT-PCR. **j** KIF4A expression in TNBC cell lines was determined by western blot (left) and quantified (right). **k** KIF4A expression in 57 pairs of TNBC tissues and normal adjacent tissues. **l** OS curves for 240 TNBC patients. **m** DFS curves for 240 TNBC patients. **P < 0.01

To validate whether circKIF4A sponges miR-375 and liberates the expression of its downstream target, we searched TargetScan for potential target genes of miR-375, and KIF4A was predicted (Fig. 4a). A subsequent luciferase reporter assay revealed decreased luciferase intensity after cotransfection of miR-375 mimics and wild type luciferase reporter, while the mutated luciferase reporter exerted no such effect (Fig. 4b). Moreover, increased luciferase intensity was observed after cotransfection of the wild type luciferase promoter and a miR-375 inhibitor (Fig. 4b). Additionally, miR-375 could suppress KIF4A expression, while a miR-375 inhibitor increased KIF4A expression (Fig. 4c and d), which indicates that KIF4A might be regulated by miR-375.

Next, a RIP assay on Ago2 showed that circKIF4A, KIF4A and miR-375 were mainly enriched to Ago2 (Fig. 4e), which indicates that circKIF4A and KIF4A are recruited to an Ago2-related RISC where they interact with miR-375. In addition, the knockdown of circKIF4A decreased the enrichment of Ago2 to circKIF4A, while it increased the enrichment of Ago2 to KIF4A (Fig. 4f), which indicates that circKIF4A could function as a ceRNA and compete with KIF4A to bind miRNAs. Furthermore, circKIF4A knockdown led to decreased KIF4A expression, while transfection with a miR-375 inhibitor reversed this decrease (Fig. 4g and h), which indicates that circKIF4A sponges miR-375 to regulate KIF4A expression.

Next, we explored KIF4A expression in TNBC cell lines and tissues and found that KIF4A was overexpressed (Fig. 4i - k). Then, we investigated the clinical impact of KIF4A on TNBC and found that KIF4A was positively associated with lymph node metastasis and TNM stage (Table 2), which indicates that KIF4A plays a vital role in TNBC progression. Kaplan-Meier survival analysis also revealed that KIF4A expression was positively associated with poorer survival of TNBC (Fig. 4l and m).

**Table 2:** Association between KIF4A and the clinicopathological characteristics of TNBC

Variables	Cases (*n* = 240)	KIF4A		*P* value
		Low (*n* = 130)	High (*n* = 110)	
Age (years)				0.257
< 50	136	78 (57.4%)	58 (42.6%)	
≥ 50	104	52 (50.0%)	52 (50.0%)	
Menopause				0.428
no	144	81 (56.3%)	63 (43.8%)	
yes	96	49 (51.0%)	47 (49.0%)	
Tumor Size				0.070
≤ 2.0 cm	66	42 (63.6%)	24 (36.4%)	
> 2.0 cm	174	88 (50.6%)	86 (49.4%)	
Lymph node Metastasis				<0.001*
No	123	81 (65.9%)	42 (34.1%)	
Yes	117	49 (41.9%)	68 (58.1%)	
TNM Stage				<0.001*
I-II	187	113 (60.4%)	74 (39.6%)	
III-IV	53	17 (32.1%)	36 (67.9%)	

*P < 0.05, statistically significant

## Discussion

Numerous circRNAs have been found to be deregulated and to act as oncogenic stimuli or tumor suppressors in various cancers. For instance, circFoxo3 has been reported to promote cell apoptosis and inhibit angiogenesis and cell cycle progression in cancer [8, 9], while ciRS-7 promotes cell cycle progression by enhancing the EGFR/RAF1/MAPK pathway [10]. Here, we reanalyzed circRNAs expression in TNBC and found that circKIF4A was significantly upregulated and positively associated with tumor size, lymph node metastasis, TNM stage and worse outcome of TNBC patients. Subsequent experiments revealed that circKIF4A regulated TNBC cell proliferation and migration. These results revealed that circKIF4A may act as a prognostic biomarker and therapeutic target for TNBC.

Increasing evidence shows that circRNAs are important posttranscriptional regulators. Due to the abundance, stability and the potential number of MREs they contain, circRNAs are effective miRNA sponges [2]. circHIPK3 is a miR-124 sponge and silencing circHIPK3 significantly inhibits cell growth [11]. circMTO1 sponges miR-9 to promote p21 expression and suppress cancer progression [12]. Yu J et al. indicated that cSMARCA5 sponges miR-17 and miR-181b to inhibit cancer proliferation and migration [13]. These findings reveal that circRNAs could act as miRNA sponges and thereby regulate cancer process. But no preclinical reports on circRNAs as targets or therapeutic vectors for cancer treatment have been published thus far [14].

Recently, miR-375 has been reported as a tumor suppressor that is significantly downregulated in multiple cancer types [15]. In esophageal carcinoma, miR-375 inhibits tumor growth and metastasis through the inhibition of IGF1R [16]. In gastric cancer, miR-375 is markedly downregulated and inhibits cell proliferation by targeting JAK2 [17]. In hepatocellular carcinoma, miR-375 targets AEG-1 to suppress cell growth [18]. In breast cancer, miR-375 could sensitize resistant cells to tamoxifen and partly reverse EMT [19]. Consider the vital function of miR-375 in cancer, developing a miR-375-based therapy is encouraging for cancer treatment.

KIF4A (kinesin family member 4A) has been identified as an oncogene that is overexpressed in several malignancies including breast cancer. High KIF4A expression is significantly correlated with poor prognosis in multiple cancers. KIF4A is essential to cancer progression and therefore has the potential to be a prognostic biomarker and therapeutic target. Huang Y et al. found that KIF4A is upregulated and correlated with poorer survival of hepatocellular carcinoma [20]. And elevated levels of KIF4A are associated with poor survival of breast cancer and that knockdown of KIF4A strongly suppresses cell proliferation and induces apoptosis [21]. Moreover, the inhibition of KIF4A suppresses cell growth in lung cancer [22].

Here, we explored the potential regulatory mechanisms of circKIF4A in TNBC and found that circKIF4A regulated the expression of KIF4A via sponging miR-375 to exert its regulatory functions in TNBC. The circKIF4A-miR-375-KIF4A axis regulates TNBC progression via the ceRNA mechanism.

## Conclusions

In summary, circKIF4A is significantly upregulated and is positively associated with worse outcomes in TNBC patients. circKIF4A, which could regulate TNBC cell proliferation and migration, also regulates KIF4A expression via sponging miR-375 to exert its regulatory functions in TNBC. The circKIF4A-miR-375-KIF4A axis regulates TNBC progression via the competing ceRNA mechanism. circKIF4A may therefore serve as a prognostic biomarker and therapeutic target for TNBC.

## Additional file

Additional file 1: **Table S1.** The sequences of siRNAs and primer sequences for qRT-PCR used in this study. (XLSX 12 kb)

## Abbreviations

ceRNAs: Competitive endogenous RNAs
circRNAs: Circular RNAs
RIP: RNA immunoprecipitation
TNBC: Triple-negative breast cancer
